# A compact silicon grating coupler based on hollow tapered spot-size converter

**DOI:** 10.1038/s41598-018-20875-3

**Published:** 2018-02-07

**Authors:** Md Asaduzzaman, Masuduzzaman Bakaul, Efstratios Skafidas, Md Rezwanul Haque Khandokar

**Affiliations:** 1 Department of Electrical and Electronic Engineering, The University of Melbourne, Parkville, VIC 3010 Australia; 2 Data61/CSIRO (Commonwealth Scientific and Industrial Research Organization), 343 Royal Parade, Parkville, VIC 3052 Australia; 3 School of Engineering, Monash University Malaysia, 47500 Bandar Sunway, Selangor Malaysia

## Abstract

A new compact silicon grating coupler enabling fibre-to-chip light coupling at a minimized taper length is proposed. The proposed coupler, which incorporates a hollow tapered waveguide, converts the spot-size of optical modes from micro- to nano-scales by reducing the lateral dimension from 15 µm to 300 nm at a length equals to 60 µm. The incorporation of such a coupler in photonic integrated circuit causes a physical footprint as small as 81 µm × 15 µm with coupling efficiency and 3-dB coupling bandwidth as high as 72% and 69 nm respectively.

## Introduction

Although high refractive index (RI) contrast in silicon-on-insulator (SOI) platforms enables multifunctional submicronic integration of photonic components in a single chip, it leads to the challenges of interfacing them with standard single mode fiber (SMF)^1^. Such issue arises due to dimensional mismatch between nano-scale waveguide devices and micro-scale SMF, typically in the order of 10^−3^, which results in excessive modal loss while coupling directly. Therefore, the requirement of efficient coupling becomes indispensable to minimize the coupling loss. Among various coupling methods, grating coupler (GC), while compared with widely investigated facet coupler^2–5^ involving post-CMOS fabrication complexities of dicing and polishing, has attracted a lot of attention due to its high compatibility with standard CMOS fabrication processes. GC also offers the flexibility to place it on any position of the photonic chip leveraging wafer level testing in mass manufacturing processes^6,7^. However, GC’s inherent characteristics in supporting propagation in multiple directions is causing a major hindrance to attain sufficient directionality for desired coupling efficiency (CE) and/or coupling bandwidth (CBW). To achieve higher directionality in GC, bottom mirrors based on either metal^8–10^ or distributed Bragg reflector (DBR)^11^ were incorporated, which usually require customized (non-standard) wafers or CMOS processes. An alternative CMOS compatible silicon nitride (Si_3_N_4_) GC with bottom Si grating reflector was proposed^12^ which, however, still requires multiple etching and lithographic processes. Apart from the approaches with bottom mirrors, directionality were also improved by adding interleaved trenches^13^ and anti-phase reflection coatings^14^ with limited success in improving CE and CBW.

Mode-shape difference between fiber and waveguide modes, and back reflection at the point of incidence also curtail CE and CBW of GC severely. Whilst mode-shape can be improved by using apodized grating structure^15–17^ or fully etched photonic crystals^18^ to produce Gaussian like profile, controlling back reflection requires further engineered structures such as Si overlay^19^ or shallow-etched grating structure^20^ at the expense of additional complexity in lithography and/or etching processes. Another noteworthy drawback of GC is the polarization dependency, which is caused by uneven diffraction in transverse electric (TE) and transverse magnetic (TM) modes. Polarization diversity 1D and 2D grating structures, with their relative merits and demerits^21–23^, were proposed to attain similar CE/CBW in both TE and TM modes^24,25^. Perfectly vertical GC is another area of active investigation since the recent past to realize cost-effective photonic packaging^26–29^.

Although, in general, issues regarding GC have been investigated widely, the challenge of interfacing nano-scale waveguide devices with SMF still remains, as most of the GC structures convert only the vertical dimensions of the waveguides to nano-scales, leaving the lateral dimensions unaltered. Therefore, to enable complete interfacing, GC structures must be adiabatically tapered down to nano-scales, which demand further analysis of CE and CBW. Moreover, as the efficiency of a tapered waveguide largely relies on the length of adiabatic transition, an undesirably large footprint of the GC structures in PIC is inevitable.

To realize smaller footprint GC for a PIC, various techniques have been proposed in the recent past. Focusing GC structure is one of such initiatives, where geometry of the gratings is cylindrical with a common focal point instead of straight lines^30–32^. Smaller footprint GCs with partially overlay dual taper^33^ and micrometric SOI rib waveguide^34^ were also proposed. Among them although focusing grating structure offers relatively smaller footprints, it usually happens at the cost of compromised CE and CBW that restrict its application in many modern technologies including optical interconnects, dense-wavelength-division-multiplexing, frequency-comb generation and so forth. In this report, we propose a new compact GC structure by replacing the conventional tapered waveguide with a hollow tapered waveguide (HTW). The proposed HTW reduces the lateral width of the grating waveguide from 15 micrometer (µm) to 300 nanometer (nm) at a length of 60 µm, keeping the height of the waveguide all through 220 nm.

The work reported in this article is organized as follows: Section II describes the modelling environment and approaches used to extract the background parameters for the proposed GC. Section III describes the proposed HTW optimized for lateral matching with nano-scale waveguide devices. Section IV presents the results obtained from simulation and performance characterization of the HTW under discussion. Section V performs a comparison of the proposed HTW with conventional and inverse tapered waveguides. Possible manufacturing process and the associated tolerances are discussed in section VI. Finally, the report is ended with concluding remarks in Section VII.

## Design Methodology and Background Parameter Extraction for the Proposed GC

**Figure 1:**
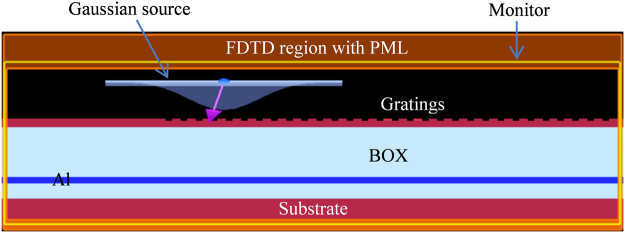
Simulation environment setup using Lumerical FDTD Solutions.

The design exercise performed in this investigation rely on widely used Finite Difference Time Domain (FDTD) method, which is a numerical analysis technique used for modelling computational electromagnetics by solving Maxwell’s equations. Although, in real, GC is a computation-intensive 3D problem requiring huge computer memory for modelling, a 2D approximation of the structure can also mimic the same functionality with sufficient accuracy, as typical width of a GC (e.g. 15 µm) is much larger in compare to its height (e.g. 220 nm) and operating wavelength (e.g. 1550 nm)^35^. Theoretically, FDTD method performs its best for grid spacing (Δ_x_, Δ_y_, and Δ_z_) approaching towards zeros. But, in practice, Δ is also perceived as a compromise between cost and complexity, and therefore, chosen to find the right balance among computational cost and accuracy. For the analysis under discussion, the modelling environment was created by using Lumerical’s FDTD solution with grid spacing as small as Δ_x_ = Δ_y_ = 10 nm, which is sufficient to maintain higher accuracy. Reflections at the boundary of the structure was eliminated by using built-in perfectly matched layer (PML)^36^. The modelling also involved parameter sweeping module where parameters for best performance can be obtained and used to update other parameters by employing Particle Swarm Optimization (PSO)^37,38^ for optimum performance. The simulation environment created for the proposed GC is shown in Fig. 1.

**Figure 2:**
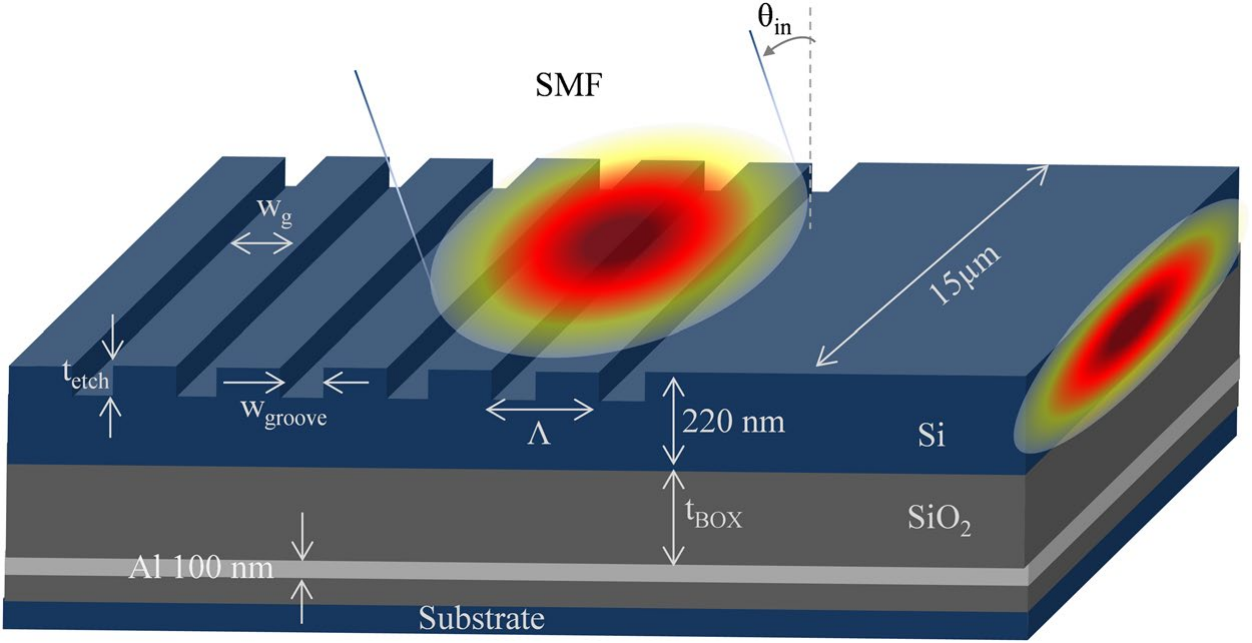
Geometry of the grating coupler structure (drawn not in scale).

To extract the background parameters for the proposed GC, a grating structure enabling typical vertical matching between SMF and nano-scale waveguide was designed, the schematic of which is shown in Fig. 2. It consists of 2 µm thick SiO_2_ layer as BOX with 220 nm Si on top to ensure single mode operation. The BOX is incorporated with an Aluminum (Al) layer of thickness of 100 nm as reflector which recaptures the light wasted through the BOX due to downward radiation^9^. The thickness of the SiO_2_ between top Si layer and Al layer is optimized as *t*_*BOX*_ = 1.6 µm that causes constructive interference among guided and reflected waves^39^. The gratings are written on top Si layer for which design parameters were are calculated by using the following grating equation:

1 $${\rm{\Lambda }}=\frac{\lambda }{{n}_{eff}-{n}_{top}.\,\sin \,{\theta }_{in}}$$

where Λ is the grating period, λ is the wavelength of incident light, *n*_*eff*_ is the effective refractive index (RI) experienced by the optical modes, *n*_*top*_ is the RI of the cladding on top of Si layer, and *θ*_*in*_ is the angle of

incidence with normal to the direction of incidence causing a slightly tilted positioning of SMF to minimize second order reflections. For the fundamental mode, *n*_*eff*_, which was calculated by using Full-Vectorial Mode Solver, was found to be 2.23. The targeted wavelength was set at λ = 1550 nm from which grating period was calculated as Λ = 636 nm for *n*_*top*_ = 1 (air) and *θ*_*in*_ = −12^0^. Shown in Fig. 1, a TE polarized Gaussian type optical source with a mode-field diameter comparable to that of SMF (approx. 10 µm) is applied on the surface of the grating. The width of the grating waveguide is chosen to be 15 µm for sufficient overlapping. The position of the source is also optimized using parameter sweep setup which is 1700 nm away from the leftmost end of the grating and 1500 nm above the grating surface. Initially grating depth (*t*_*etch*_) and filling factor (*ff*) were set to 50% of the height of top Si waveguide and grating period respectively, which later was optimized as 95 nm and 350 nm respectively for maximum efficiency by using PSO. As *ff* = (*w*_*g*_*/*Λ) = 0.55, groove width (*w*_*groove*_) was left to be 286 nm. Also, as the total height of top Si layer is 220 nm, a 125 nm Si base remains as a waveguide after etching 95 nm-depth gratings.

**Figure 3:**
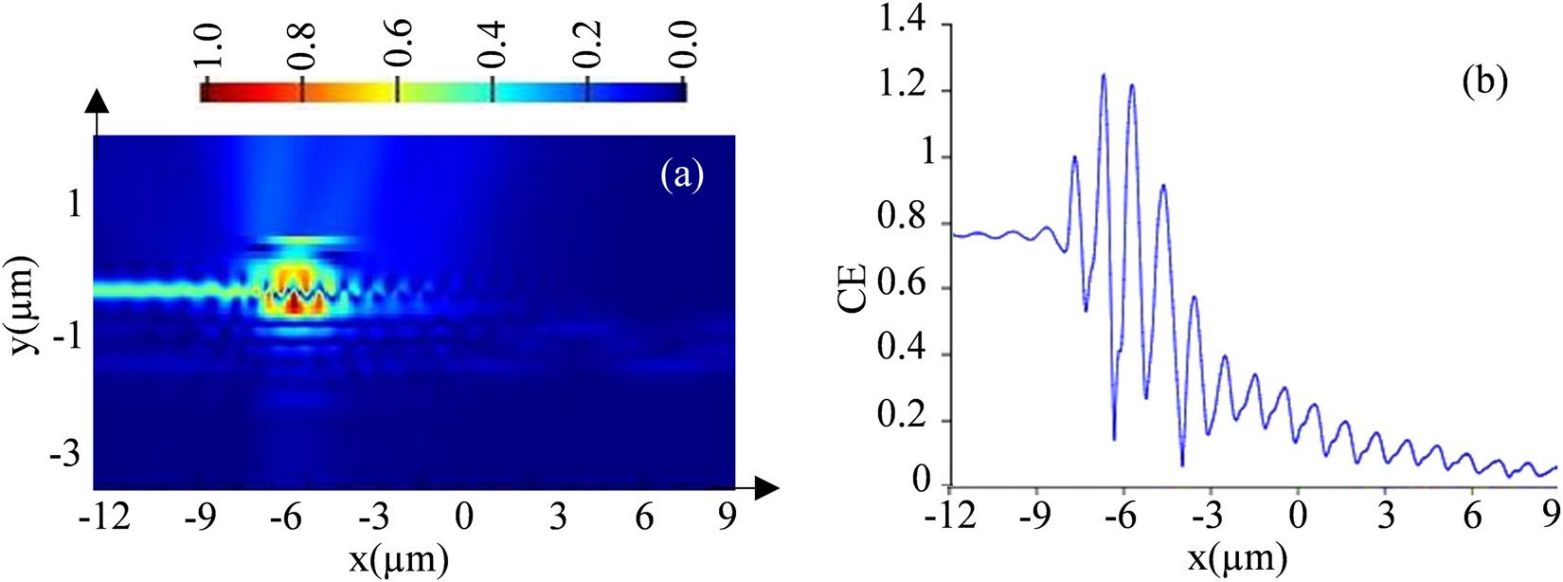
(**a**) E-field distribution along the grating structure, and (**b**) propagation of light along the structure with predicated CE.

With all these necessary parameters optimized, the E-field distribution and propagation of light along the structure are shown in Fig. 3(a,b), which were obtained by placing a power monitor across the structure. Shown in Fig. 3(b), light with a CE of 78% can be expected at the end of grating waveguide which then would be passed to the nano-scale waveguide device through the proposed HTW.

## Proposed HTW for Compact GC

**Figure 4:**
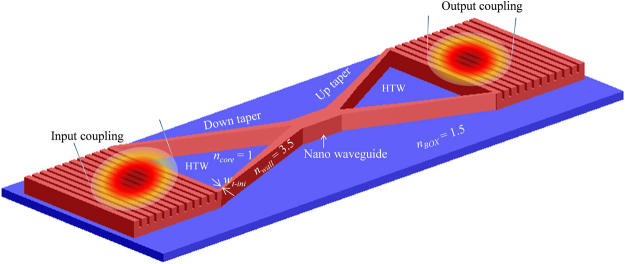
Schematic diagram of a compact GC incorporating the proposed HTW spot-size converter (drawn not to scale).

A HTW enabling lateral matching between grating waveguide and nano-scale waveguide devices with a smaller footprint is proposed and modelled by using Lumerical’s 2D FDTD solution, the related simulation environment and background parameters of which were discussed in Section II. In HTW, light is guided through a tapered hollow core where the optical modes are converted from loosely confined mode to highly confined mode. HTW is designed by inserting two Si strips one ends of which are connected with grating waveguide and other ends merge together to form a hollow core. For effective recapturing of the mode to couple to the nano-scale waveguide device, the widths of the Si strips are adiabatically increased until it supports a mode to confine within the merged Si strips. The schematic diagram of the compact GC incorporating the proposed HTW is shown in Fig. 4.

**Figure 5:**
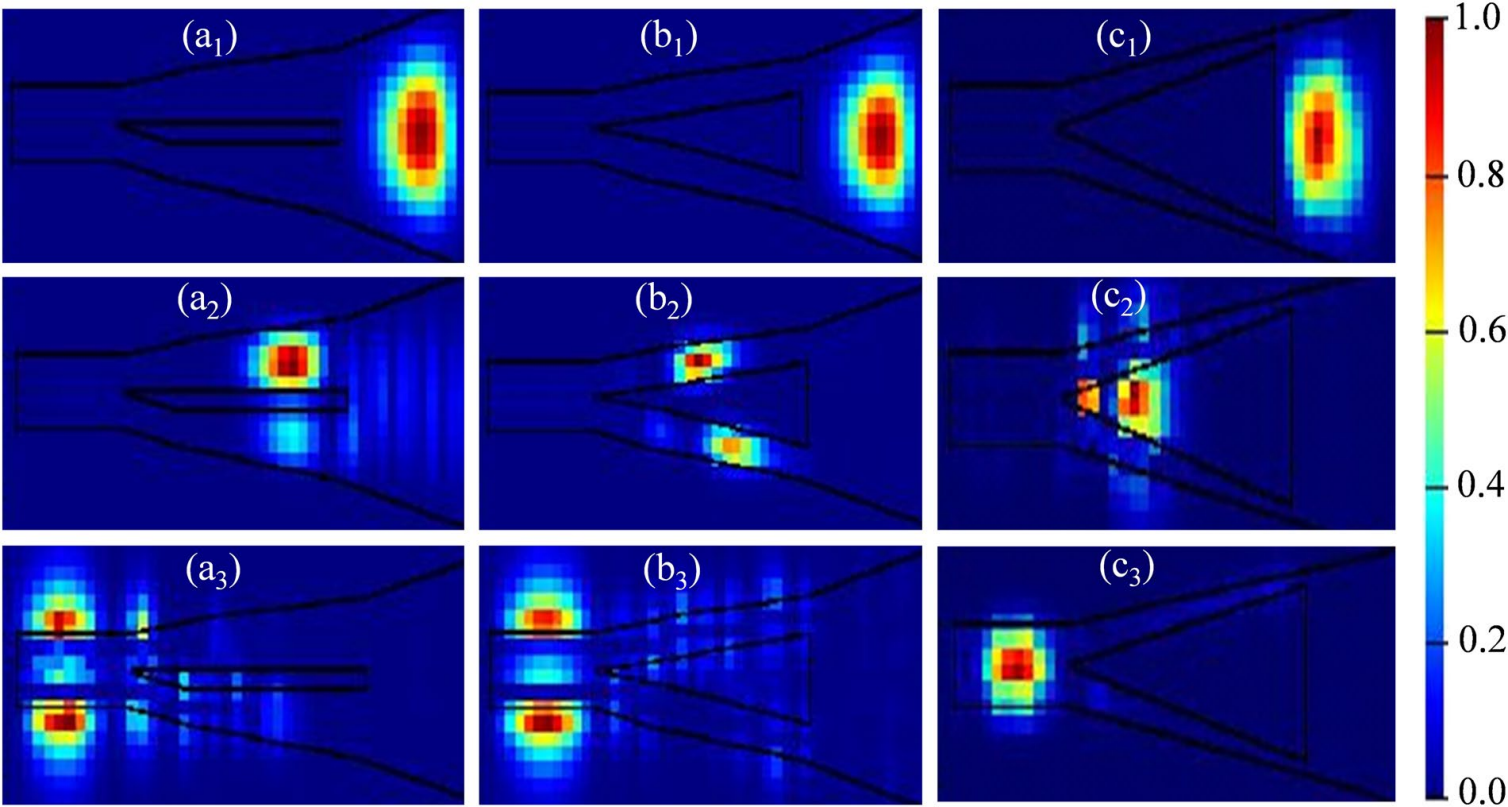
Optical modes in different locations of the hollow structured waveguide for various widths of Si strip: **(a**_**1**_**)**–**(a**_**3**_**)** for a strip-width of 500 nm, **(b**_**1**_**)**–**(b**_**3**_**)** for a strip-width of 300 nm, and **(c**_**1**_**)**–**(c**_**3**_**)** for a strip-width of 100 nm.

Optical mode from the grating waveguide is confined within the hollow air core mainly due to two physical occurrences: (i) contrast of RI between Si wall (*n*_*wall*_ = 3.5) and air core (*n*_*core*_ = 1), and (ii) the narrow width of the Si strips (*w*_*t-ini*_), which is thin enough to reject any mode inside the strip. Typical cross-section of a Si strip that fulfils the conditions for a single mode operation at 1300 to 1600 nm band is approximately 500 nm (width) ×200 nm (height)^40,41^. The height of the strips is matched with grating waveguide to 220 nm, which also satisfies the conditions for single mode operation. To find the optimum width that rejects any part of the optical mode to reside within it, HTW structure is simulated by using Finite Difference Eigenmode (FDE) solver of the Lumerical’s Mode Solutions with strip-width (*w*_*t-ini*_) setting at 500 nm, 300 nm and 100 nm, the results of which are shown in Fig. 5(a–c) respectively.

Shown in Fig. 5(a) for the strip-width of 500 nm, although the mode is fully confined within the waveguide at the beginning of HTW (Fig. 5a_1_), it appears to split and disperse gradually down the tapered waveguide (Fig. 5a_2_), and disperse completely at the narrow-end of HTW (Fig. 5a_3_), missing the coupling with the waveguide devices. Similar occurrences happen for the strip-width of 300 nm with a bit lower dispersion, as shown in Fig. 5(b_1_–b_3_). The circumstances however improve significantly for strip-widths less than 200 nm. Shown in Fig. 5(c_1_–c_3_) for the strip-width of 100 nm, mode is fully confined within the core of HTW both at the beginning and ending of the tapering, confirming the coupling with the nano-scale waveguide devices. In all cases, the length of HTW was maintained 60 µm and an adiabatic control on Si strips was applied at the narrow-end so that a mode can be fully contained.

## Performance Characterization of the Proposed HTW

**Figure 6:**
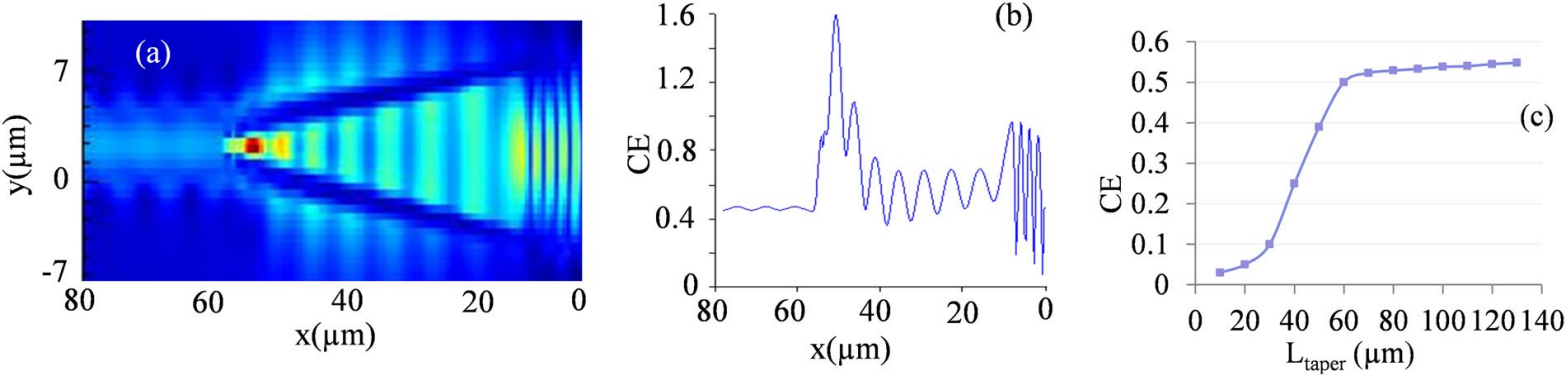
Performance characterization of the proposed HTW: (**a**) E-field distribution along the HTW structure, (**b**) estimated CE with a fixed taper length of 60 µm, and (**c**) estimated CE with respect to various taper lengths.

From the findings of previous section, it is clear that the widths of Si strips at the grating end should be around 100 nm each that need to be increased gradually at the device end for effective coupling. The distribution of E-field along the HTW and the associated CE are shown in Fig. 6(a,b). It shows that at a taper length of 60 µm, the CE estimated is only 47%. To check the influence of the taper length, the structure was further simulated with taper lengths of 20 to 140 µm and the respective estimations of CE are shown in Fig. 6(c). It confirms that CE with taper length of 60 µm is somewhat maximum and with taper lengths above 60 µm, it quickly saturates without any appreciable increment. By recalling the CE from the vertically matched grating waveguide, coupling loss in the HTW is approximately (78–47%) or 31%. This can be attributed to the modal loss at the waveguide device end where light is transported from loosely confined HTW to strongly confined nano-scale waveguide devices.

To minimize the modal loss in the proposed HTW, additional Si strips can be inserted into hollow core. Such insertion essentially converts loosely confined modes of HTW to highly confined modes by reducing the effective width of the hollow core. The optimum number of Si strips can be calculated as follows:

2 $${n}_{strip}=\frac{m-(\frac{m}{{w}_{n}}){w}_{s}}{{w}_{n}}$$

3 $$m={w}_{g}-2{w}_{s}$$

where *n*_*strip*_ is the number of Si strips, *w*_*g*_ is width of grating, *w*_*s*_ is width of strip and *w*_*n*_ is width of nano-scale waveguide device.

The spacing between adjacent Si strips (*w*_*H*_) in the HTW can be calculated as:

4 $${w}_{H}=\frac{(\frac{m}{2}-({w}_{s}\cdot \frac{{n}_{strip}-1}{2}))}{(\frac{{n}_{strip}-1}{2})}$$

**Figure 7:**
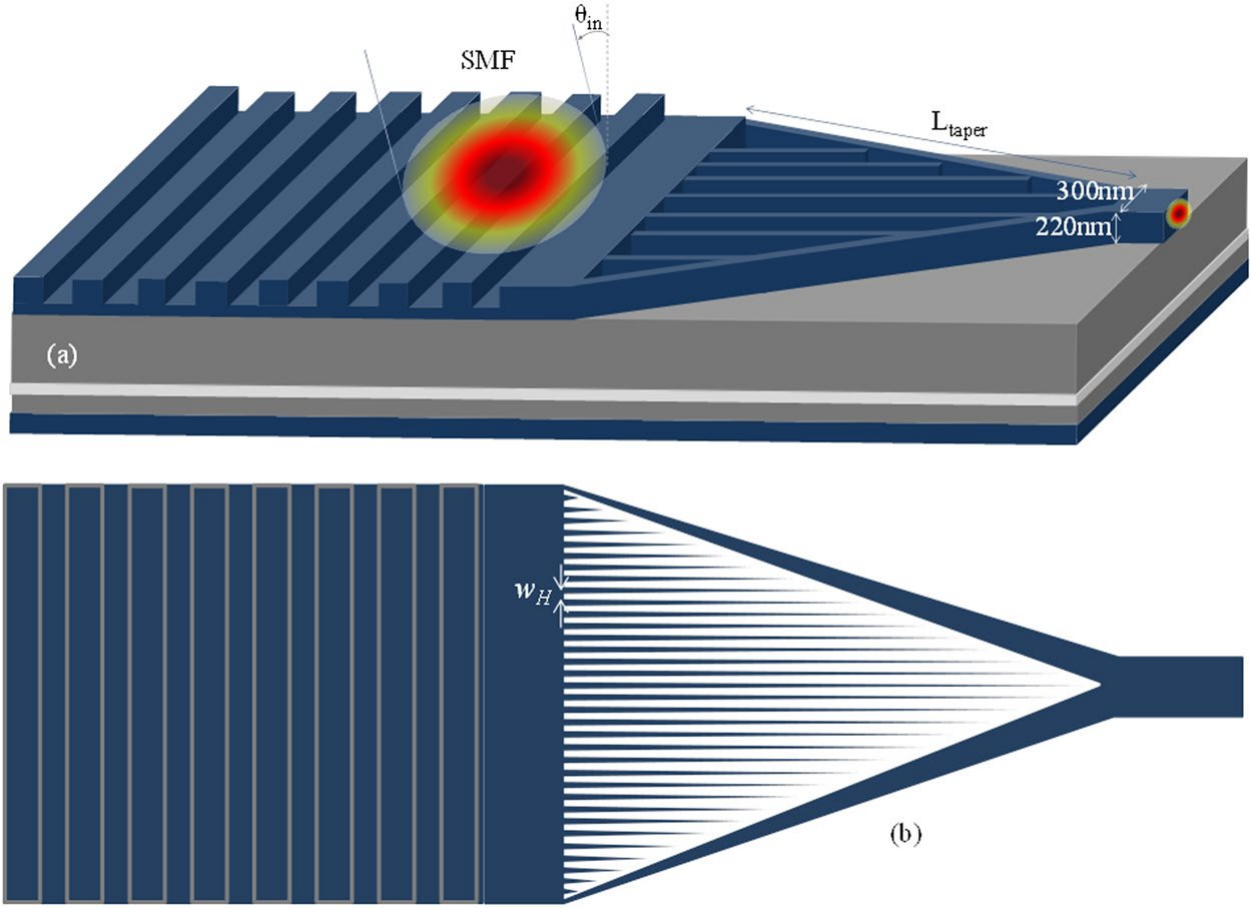
**(a)** 3D view of the GC incorporating proposed HTW (for clear view only few Si strips are shown), **(b)** 2D top view of the full HTW core with 33 Si strips (drawn not to scale).

For the parameters of the GC/HTW under consideration, optimum number of Si strips can be calculated as *n*_*strip*_ = 32.88 or 33 by using equation (2), and the width of the hollow space can be calculated as *w*_*H*_ = 362.5 nm by using equation (4). The complete structure of the GC comprising of the HTW is shown in Fig. 7.

**Figure 8:**
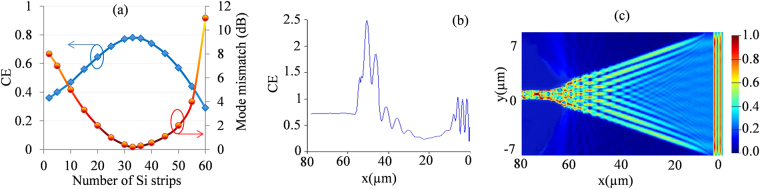
Performance of the proposed HTW after insertion of additional strips while other background parameters kept unchanged. (**a**) Changes in mode mismatch and CE with respect to the number of Si strips, (**b**) CE performance along the HTW structure with optimum 33 Si strips inserted, and (**c**) E-field distribution along the HTW structure with optimum 33 Si strips inserted.

Figure 8(a) shows the changes in mode mismatch and CE with respect to the number of Si strips in the HTW, where mode mismatch is calculated based on intermodal overlap between HTW and nano-scale waveguide device modes using FDE solver of Lumerical’s Mode solutions. Shown in Fig. 8(a), maximum theoretical CE occurs for 33 Si strips in the HTW for which mode mismatch is minimum (0.23 dB). Further increase in Si strips causes degradation in CE. This is due to the fact that adding more Si strips in HTW causes narrower hollow core width which becomes inadequate to confine a mode within itself, and as a consequence, increases the mode mismatch between HTW mode and nano-scale waveguide mode rather than reducing it. The CE performance and E-field distribution along the HTW structure with 33 Si strips are shown in Fig. 8(b,c), where it is evident that the insertion of additional strips has increased CE from 47% to 72% while kept other background parameters unchanged. Also, such insertion improved the mode confinement significantly, as can be seen from E-field distribution in Fig. 8(c).

## Performance Comparison

To quantify the improvements, proposed GC with HTW structure was compared with GCs having conventional taper (CT) and inverse taper (IT) with 60 µm taper lengths, equal to the optimum length of the proposed HTW structure. The tapers are used to couple light from 15 µm wide grating waveguide to 300 nm wide nano-scale waveguide. Height of the waveguide throughout the structure is kept 220 nm, which makes the cross-section of the nano-scale waveguide as 220 nm (height) ×300 nm (width). CE and CBW predicted for various taper structures are shown in Table 1. The results show that, for a taper length of 60 µm, conventional taper exhibits a CE of only 40% whereas the inverse taper exhibits even lower CE of 29%. Therefore, coupling loss between grating waveguide to nano-scale waveguide device is (78–40%) = 38% and (78–29%) = 49% for CT and IT respectively. In contrast, the proposed GC comprising of HTW with 33 Si strips exhibits CE up to 72% by reducing the tapering loss significantly.

**Table 1:** Performance comparison of the proposed GC with HTW under discussion with GCs incorporating CT and IT with taper length and nano-scale waveguide width of 60 µm and 300 nm respectively.

		GC + HTW		GC + CT	GC + IT
		Without strips	With strips		
CE	%	48	**72**	40	27
	dB	−3.18	**−1.42**	−3.97	−5.68
CBW(nm)	1-dB	39	**43**	34	22
	3-dB	62	**70**	51	43

**Figure 9:**
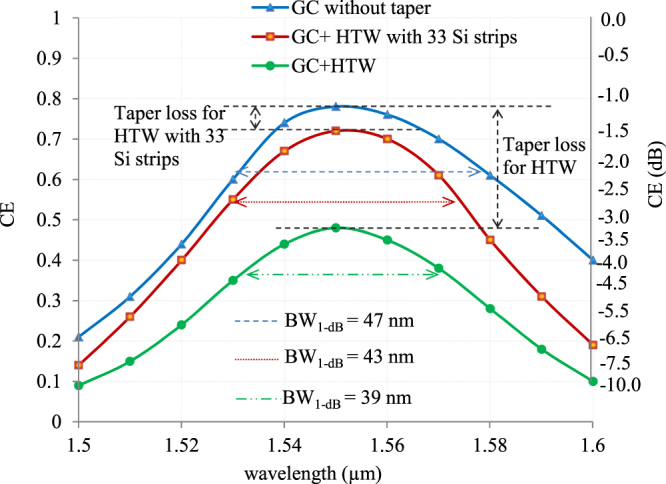
Theoretically predicted CE of the proposed GC without any taper and with HTW, both including and excluding additional Si strips, over a range of wavelengths with centre at 1550 nm.

Finally an estimation of CBWs of the designed GC without any taper and the proposed GC structure comprising of HTW with/without 33 Si strips are shown in Fig. 9. It shows that CBW does not change much with the inclusion of the proposed taper, although as expected, CE reduces by 6% for HTW with 33 Si strips, as indicated earlier.

## Fabrication Process and Tolerances

The fabrication of the structure can be started with a SOI wafer consisting 1.6 µm SiO_2_ BOX and 220 nm top Si layer. There could be two-step lithography process necessary to realize two different etch depths for gratings and HTW. In first step the gratings can be defined using electron beam lithography (EBL) technique and the pattern transfers can be realized by means of dry etching until etch depth of 95 nm. In second lithography step, the HTW with 33 Si strips need to be defined which could be achieved by using hard mask, e.g. SiO_2_ mask on top Si layer and then the top Si layer is etched until the BOX (etch depth of 220 nm) to form the proposed HTW.

**Figure 10:**
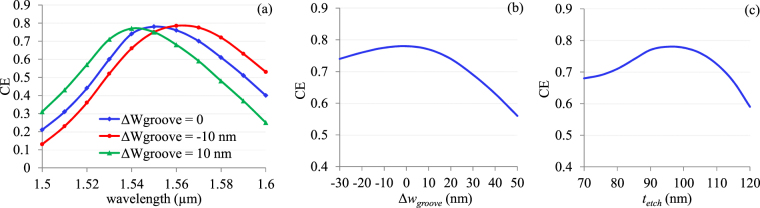
(**a**) Coupling spectra for Δ*w*_*groove*_ of +/− 10 nm, (**b**) predicted CE as function of Δ*w*_*groove*_, “0” in horizontal axis refers to no deviation of groove width which is 286 nm, (**c**) predicted CE as function of *t*_*etch*_ at the wavelength of 1550 nm.

Generally, slight variations of the designed parameters are expected in actually fabricated device. The effects of such variations on the performance of the coupler have been studied. The influence of the grating groove is shown in Fig. 10(a,b) for fixed grating period. Shown in Fig. 10(a), the coupling spectra for the groove width variation (Δ*w*_*groove*_) by +/− 10 nm from the designed width of 286 nm show that the peak of spectrum is shifted towards longer wavelength for smaller groove width while the change in opposite direction happens for larger groove width. Shown in Fig. 10(b), CE as function of Δ*w*_*groove*_ drops more for positive variation (larger grooves) than that of negative variation (smaller grooves). However, irrespective of larger or smaller groove, these variations are quite insignificant (less than 1%) for a groove width variation of +/− 10 nm. The effects of etch-depth variation is also studied and encapsulated in Fig. 10(c). It shows that CE drops more for deeper etch-depth in compare to shallow etch-depth. A CE change of less than 1% (ΔCE < 1%) is predicted for etch-depth (*t*_*etch*_) range of 90–100 nm. ΔCE will be around 4% only for the change of *t*_*etch*_ from 85 nm to 110 nm.

**Figure 11:**
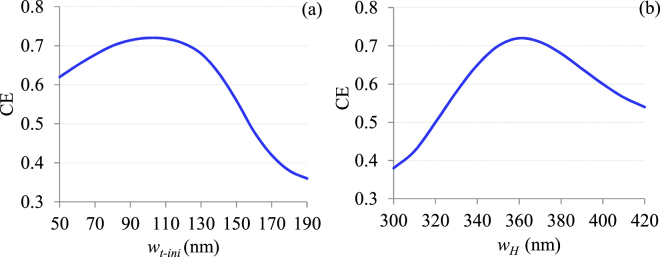
Estimation of the fabrication tolerance of HTW with respect to (**a**) *w*_*t-ini*_ and (**b**) *w*_*H*_.

The study of the fabrication tolerances was further extended to the HTW structure. First of all the influence of the initial width (*w*_*t-ini*_) of the two outer Si trips is investigated. In section III we have found that *w*_*t-ini*_ should be thinner than 200 nm to deny any mode to reside within it. So the HTW structure is simulated for *w*_*t-ini*_ from 50 nm to 190 nm as shown in Fig. 11(a). The results show that for *w*_*t-ini*_ range of 80–120 nm, CE drops only 2%, which however changes sharply for *w*_*t-ini*_ around 140 nm. This can be attributed to the fact that at such thickness mode starts escaping the hollow core and gradually penetrating into Si strips. The effects of hollow space (*w*_*H*_) was also studied (shown in Fig. 11(b)) and found that CE is more sensitive to *w*_*H*_ compare to other parameters. This is because *w*_*H*_ determines the mode mismatch between HTW and nano-scale waveguide modes. As *w*_*H*_ deviates from optimum value, mode mismatch increases and consequently drops CE. However fabrication tolerance is still well-within comfortable limit, as the change of *w*_*H*_ from 350 to 370 nm causes only 2% of ΔCE.

## Conclusion

A compact GC with HTW spot-size converter is proposed. The overall dimension of the GC is reduced to 81 µm (length) ×15 µm (width) to enable smaller footprint in PICs, while ensuring least possible coupling loss between optical fibre and nano-scale waveguide devices. Light is transported from 15 µm wide grating waveguide to 300 nm wide Si nano-scale waveguide with a proposed 60 µm long HTW. The 2D FDTD analysis shows that, with the basic HTW structure with 2 Si strips, only 47% of incident light could be coupled to the nano-scale waveguide. To increase CE, HTW structure was improved by implanting additional Si strips into the hollow core that minimize the mode mismatch between HTW and nano-scale waveguide modes. Such HTW structure with optimum number of Si strips exhibits CE of 72% (−1.42 dB) with 1-dB and 3-dB CBW of 43 nm and 69 nm respectively. Using the proposed HTW light can be coupled from grating waveguide to nano-scale waveguide with least possible taper loss, only 6% estimated in this case. The performance of the proposed HTW structure is compared with conventional and inverse tapers for similar taper lengths as of HTW and found that the proposed HTW structure shows superior performance over conventional and inverse tapers. The proposed structure is fully CMOS compatible and can be fabricated based on standard lithography and etching process. The fabrication tolerance also investigated and found that the device possess remarkable high tolerant to the fabrication errors. The results are comparable with most compact designs in literature taking the size, coupling efficiency and bandwidth into account. The coupling efficiency can further be improved by reducing back reflection and implementing apodized gratings to achieve higher mode matching between fibre and waveguide modes.
